# Editorial: Recent advances in synthesizing and utilizing nitrogen-containing heterocycles

**DOI:** 10.3389/fchem.2024.1421449

**Published:** 2024-05-10

**Authors:** Takashi Ohshima, Hsyueh-Liang Wu, Hyun-Joon Ha

**Affiliations:** ^1^ Graduate School of Pharmaceutical Sciences, Kyushu University, Fukuoka, Japan; ^2^ Department of Chemistry, National Taiwan Normal University, Taipei, Taiwan; ^3^ Department of Chemistry, Hankuk University of Foreign Studies, Yongin, Republic of Korea

**Keywords:** aziridine, azetidine, pyrrolidine, imidazolidine, pyrazole, triazoles, piperidine, pyridine

Exploring the vast landscape of nitrogen-containing heterocycles, commonly known as aza-heterocycles, reveals their ubiquitous presence in natural products, pharmaceuticals, agrochemicals, and functional materials. Consequently, synthesizing these compounds has garnered significant attention, driven by the need to develop more efficient methods for their preparation and utilization as versatile building blocks. The importance of nitrogen-containing heterocycles has increased due to their expanding role as ligands in transition-metal catalysis and organocatalysis. Despite extensive studies into their synthesis and applications, ongoing demand remains for more efficient and general preparation methods to diversify the structural backbones and improve their biological activities. Moreover, their usage as ligands and catalysts is continuously being investigated in various chemical reactions.

This Research Topic, “Recent Advances in Synthesizing and Utilizing Nitrogen-Containing Heterocycles,” curated by Professors Ohshima, Wu, and Ha, features a Research Topic of groundbreaking research, illuminating innovative synthetic methods, strategies for selectivity and reactivity modulation, and diverse applications in functional molecule design. This editorial presents ten original research articles and one perspective piece, highlighting essential findings and their broader implications in the field of heterocyclic chemistry, offering promising insights into the future of this vibrant scientific area.

The Innovative Synthetic Methods section showcases pioneering strategies for constructing nitrogen-containing heterocycles. Murakami et al. present a copper-catalyzed method for synthesizing imidazolidine and imidazolidinone by reacting aziridines with imines and isocyanates, respectively. This approach involves transforming 3-membered heterocyclic rings into 5-membered ones, yielding a diverse array of 2-substituted imidazolidines and substituted imidazolidinones with high functional group compatibility. Lee et al. introduce a novel method for constructing functionalized dihydropyridinone rings via the annulation of an amide tethered with an alkyne moiety at the α-carbon. This process includes the formation of O-silyl N,O-ketene acetal and silver-mediated addition, proving a new route for the total synthesis of phenanthroindolizidine and phenanthroquinolizidine alkaloids. Kim et al. descrive a new single-atom deletion strategy for the late-stage conversion of alkaloids, employing oxidative ring contraction followed by chemoselective reduction to convert the 6-membered piperidine moiety of (allo)securinine into a 5-membered pyrrolidine, facilitating access to (allo)norsecurinine.

In the Selectivity and Reactivity Control domain, it is demonstrated that modifications to the catalyst, reaction conditions, or substrate structure can significantly enhance selectivity and reactivity, suggesting a new control strategy in heterocyclic synthesis. Kuriyama et al., focusing on La(OTf)3-catalyzed reactions, illuminate the efficiency and versatility of modern synthetic strategies. Their method utilizing La(OTf)3-catalyzed intramolecular regioselective aminolysis method to convert cis-3,4-epoxy amines into highly strained 4-membered ring azetidines over 5-membered ring pyrrolidines. This approach achieves high yields even with acid-sensitive and Lewis basic functional groups, marking a significant advancement in synthetic organic chemistry. Srivastava and Ha develop an innovative approach for constructing nitrogen-containing heterocycles via aziridine ring-opening reactions. In this reaction, the regioselectivity depends on the functional groups of the alkyl substituents. Employing aziridine rings substituted with ketone or silylated hydroxy group as substrates has proven to efficiently facilitate ring-opening and subsequent cyclizations, converting 3-membered heterocyclic rings into 5- or 6-membered ones. Noda et al. investigate the reactivity of rhodium alkyl nitrenes derived from substituted hydroxylamine precursors for synthesizing 5-membered pyrrolidines, focusing on modulating the regioselectivity between benzylic and tertiary C–H bonds by adding of Brønsted acids or by modifying oxygen substituents. Their findings deepen the understanding of metallonitrene structures and provide valuable insights for the selective synthesis of N-heterocycles. This study underscores the profound impact of precursor structures and additives on nitrene reactivity, paving the way for substrate-controlled synthesis, which is crucial for medicinal chemistry and drug development. Kim et al. develop a novel and highly efficient strategy for the C4-selective (hetero)arylation of pyridines using N-aminopyridinium salts. This method overcomes the poor site-selectivity often encountered in conventional methods by using N-aminopyridinium salts instead of pyridine as substrates. This metal-free method proceeds at room temperature with a base, eliminating the need for catalysts or oxidants. It allows the incorporation of various electron-rich (hetero)aryl groups onto pyridines, facilitating the synthesis of valuable C4-(hetero)aryl pyridine derivatives, crucial in agrochemicals, pharmaceuticals, and functional materials. In a complementary contribution, Choi et al. explore the compatibility of various functional groups in the hydrazinolysis of amides with ammonium salts. The resulting acyl hydrazide can be readily converted into the corresponding ester through acylpyrazole. Utilizing a Functional Group Evaluation (FGE) kit comprising 26 additives, including nitrogen-containing heterocycles like imidazole and indole, this study rapidly assesses the compatibility of functional groups crucial for drug discovery research. Furthermore, this innovative research unveils the positive effects of carboxylic acid additives, suggesting the utility of this evaluation kit in developing new reaction systems.

The Applications for Functional Molecules segment transcends the boundaries of traditional synthesis by exploring the applications of nitrogen-containing heterocycles across various scientific fields.

Further expanding the synthetic repertoire, Namioka et al. present an efficient method for preparing organomagnesium intermediates with protected azido groups, utilizing Amphos for azide protection. This technique facilitates the synthesis of diverse functionalized azides and their subsequent conversion into a wide array of 1,2,3-triazoles through click reactions. This advancement contributes significantly to synthetic organic chemistry, pharmaceutical sciences, and materials chemistry by providing a versatile approach to synthesize azides and triazoles, pivotal as synthetic intermediates and bioactive compounds. Mohamadpour et al. introduce a groundbreaking, green photosynthesis method for synthesizing 3,4-dihydropyrimidin-2-(1H)-one/thione derivatives which exhibit widespread biological applications such as antihypertensive, antiviral, antitumor, antibacterial, α-1a-antagonism, antioxidant, and anti-inflammatory actions, from aryl aldehydes, β-ketoesters, and urea/thiourea. This method employs a novel halogenated dicyanobenzene-based photosensitizer, 3DPAFIPN, as a donor-acceptor photocatalyst triggered by visible light. Utilizing blue LED technology enables a sustainable, energy-efficient reaction process in an ethanol medium at room temperature. The perspective offered by Ha brings to light the practicality and environmental benefits of using proline-derived organocatalysis and pot-economical synthesis. The synthesis of (−)-quinine serves as a prime example of overcoming synthetic challenges through organocatalysis, showcasing the method’s potential in streamlining the environmentally benign production of complex organic compounds. The discussion includes various organocatalysis techniques and their success in enhancing reaction conditions, emphasizing the significance of organocatalysts in modern synthetic organic chemistry.

This Research Topic showcases the dynamic and ever-evolving landscape of research on nitrogen-containing heterocycles and lays the groundwork for future investigations. It is a testament to the collaborative spirit of the scientific community, driven by a shared commitment to unravel the complexities of chemistry for the advancement of knowledge and society.

We extend our deepest appreciation to all the contributors, whose dedication and groundbreaking work enrich our collective understanding and pave the way for future innovations. Their diverse insights and findings reflect the multifaceted nature of chemistry’s quest to harness the potential of nitrogen-containing heterocycles, promising new avenues for discovery and application.

